# A simple, clinically applicable motor learning protocol to increase push-off during gait: A proof-of-concept

**DOI:** 10.1371/journal.pone.0245523

**Published:** 2021-01-19

**Authors:** Michaël Bertrand-Charette, Jens Bo Nielsen, Laurent J. Bouyer

**Affiliations:** 1 Center for Interdisciplinary Research in Rehabilitation and Social Integration (CIRRIS), Quebec City, Quebec, Canada; 2 Department of Neuroscience, University of Copenhagen, Copenhagen, Denmark; 3 Department of Rehabilitation, Faculty of Medicine, Laval University, Quebec City, Quebec, Canada; Baylor College of Medicine, UNITED STATES

## Abstract

**Objective:**

Task-specific training is often used in functional rehabilitation for its potential to improve performance at locomotor tasks in neurological populations. As push-off impairment are often seen with these patients, this functional approach shows potential to retrain gait overground to normalize the gait pattern and retrain the ability to improve gait speed. The main objective of this project was to validate, in healthy participants, a simple, low-cost push-off retraining protocol based on task-specific training that could be implemented during overground walking in the clinic.

**Methods:**

30 healthy participants walked in an 80-meter long corridor before, during, and after the application of an elastic resistance to the right ankle. Elastic tubing attached to the front of a modified ankle-foot orthosis delivered the resistance during push-off. Relative ankle joint angular displacements were recorded bilaterally and continuously during each walking condition.

**Results:**

On the resisted side, participants presented aftereffects (increased peak plantarflexion angle from 13.4±4.2° to 20.0±6.4°, p<0.0001 and increased peak plantarflexion angular velocity from 145.8±22.7°/s to 174.4±37.4°/s, p<0.0001). On the non-resisted side, aftereffects were much smaller than on the resisted side suggesting that the motor learning process was mainly specific to the trained leg.

**Conclusion:**

This study shows the feasibility of modifying push-off kinematics using an elastic resistance applied at the ankle while walking overground. This approach represents an interesting venue for future gait rehabilitation.

## Introduction

After neural injury, gait control is often compromised. Reduced push-off force output is one of the most prevalent gait impairments, and correlates to a reduced gait speed [1–5], limiting patients in their mobility and activities of daily living. After conventional rehabilitation, gait speed only partially recovers [6–8], and push-off force output remains sub-optimal. It is therefore warranted to improve the retraining of push-off force output in these populations in order to reduce maladaptive compensations and retrain the ability to modulate/increase gait speed.

Experimentally controlled error-based motor learning, where an externally applied perturbation is used to train the emergence of new motor programs, has been proposed as a potential protocol for training motor recovery after injury [9]. Considering that the neural control of gait results from the interaction between voluntary commands, involuntary rhythmic movement generators, and complex phase-dependent sensorimotor integration (see Barthélemy et al., 2011 & Grey et al., 2013 for reviews [10,11]), it is impractical to use purely explicit learning protocols during rehabilitation. Error-based motor learning therefore represents an approach of particular interest for gait retraining, both from its implicit nature [9] and its task-specificity [12–16].

In neurologically impaired populations, error-based motor learning protocols have shown the capacity to improve overall walking ability [17–22]. At the ankle joint specifically, results are more mitigated. For foot dorsiflexors during the swing phase, error-based motor learning was effective. This is an important finding, as improper activation of these muscles has been associated with increased risk of fall [23]. The generalizability of these studies may be limited however, as they were performed on motorized treadmills, a situation that does not represent the real-life situations of patients. When assessed, the transfer of treadmill-induced aftereffects to overground walking is unfortunately incomplete [22,24].

For ankle plantarflexors, the muscles responsible for modulating gait velocity in healthy and impaired populations [4,25,26], error-based motor learning has been less successful. Indeed, in a study using a robotized ankle-foot orthosis, Noel et al. [27] were not able to produce aftereffects (a manifestation of motor learning) during the push-off phase of gait, the moment where ankle plantarflexors are at their maximal activation. Part of this inability to modify the locomotor pattern may be related to the fact that the study was here again performed on a treadmill.

While recent advances in robotic orthoses and exoskeletons have made it possible to apply controlled perturbations during gait in the laboratory setting to induce error-based motor learning [28–32], the majority of them are cumbersome, expensive and complex to use, making them impractical for standard clinical use [33,34]. This limits large-scale implementation, and hence reduces substantially the possibility of having an impact on patients’ quality of life.

Simpler potential solutions that may be more suitable for clinical use should be studied. For example, elastic-based perturbations that can induce error-based motor learning during gait [35,36] would be an interesting low-cost, low maintenance alternative to robotic devices for the clinical setting. Furthermore, considering the limited transfer of learning from treadmill training to overground walking, overground training should also be prioritized.

As retraining of ankle plantarflexors after CNS injury is important in order to regain functional gait speed and return to community ambulation [37], the primary objective of the present study was therefore to validate, in healthy participants, a simple, error-based gait retraining protocol that could be implemented during overground walking in the clinic. To do so, the protocol tested the effect of an elastic tubing resistance applied around the ankle using a modified ankle-foot orthosis. Considering the complex nature of the neural control of walking during push-off (involving interactions between positive sensory feedback and descending drive), a secondary objective of this study was to take advantage of differences in individual adaptation strategies to identify motor strategies that might be more efficient for transferring the adapted motor pattern to regular walking (measured as increases in aftereffect duration) and optimize retention over time. Based on error-based motor learning principles, we hypothesized that this simple, low-cost push-off retraining device would result in increased peak plantarflexion angle once removed.

## Materials and methods

### Participants

A convenience sample of 30 non-disabled participants (see Table 1 for demographic data) was recruited from Université Laval’s student population. They had to be naive to the task and aged between 18 and 65 years. The exclusion criteria were known history of neurological or musculoskeletal disorders that could interfere with task execution. All participants read and signed a consent form describing the experimental procedure and their involvement in the study. This protocol was approved by the local ethics committee (CIUSSS-CN #2016–578) and the experimental procedures were in accordance with the Declaration of Helsinki.

**Table 1:** Group demographic data.

Code	Sex	Footedness	Age	Height	Weight	BMI
S01	F	D	29	180	66	20.4
S02	F	D	24	168	64	22.7
S03	F	D	25	166	60	21.8
S04	F	D	23	160	51	19.9
S05	F	D	21	165	57	20.9
S06	F	D	23	160	74	28.9
S07	F	D	23	167	73	26.2
S08	M	D	21	175	84	27.4
S09	M	D	28	170	70	24.2
S10	M	D	21	168	64	22.7
S11	F	D	22	165	70	25.7
S12	M	D	25	185	77	22.5
S13	M	D	22	170	64	22.1
S14	F	D	20	172	82	27.7
S15	M	D	24	183	84	25.1
S16	M	D	25	184	77	22.7
S17	M	D	24	175	72	23.5
S18	F	D	26	165	63	23.1
S19	F	D	28	165	61	22.4
S20	F	D	24	170	70	24.2
S21	F	D	24	168	61	21.6
S22	M	D	22	174	68	22.5
S23	F	D	24	166	65	23.6
S24	F	D	29	170	71	24.6
S25	M	D	21	184	75	22.2
S26	M	D	25	175	79	25.8
S27	M	D	27	180	76	23.5
S28	M	D	24	178	79	24.9
S29	M	D	23	168	61	21.6
S30	M	D	27	175	73	23.8
Mean			24.1	171.7	69.7	23.6
SD			2.5	7.0	8.2	2.2
Range			20–29	160–185	51–84	19.6–28.9

### Elastic resistance

**Fig 1:**
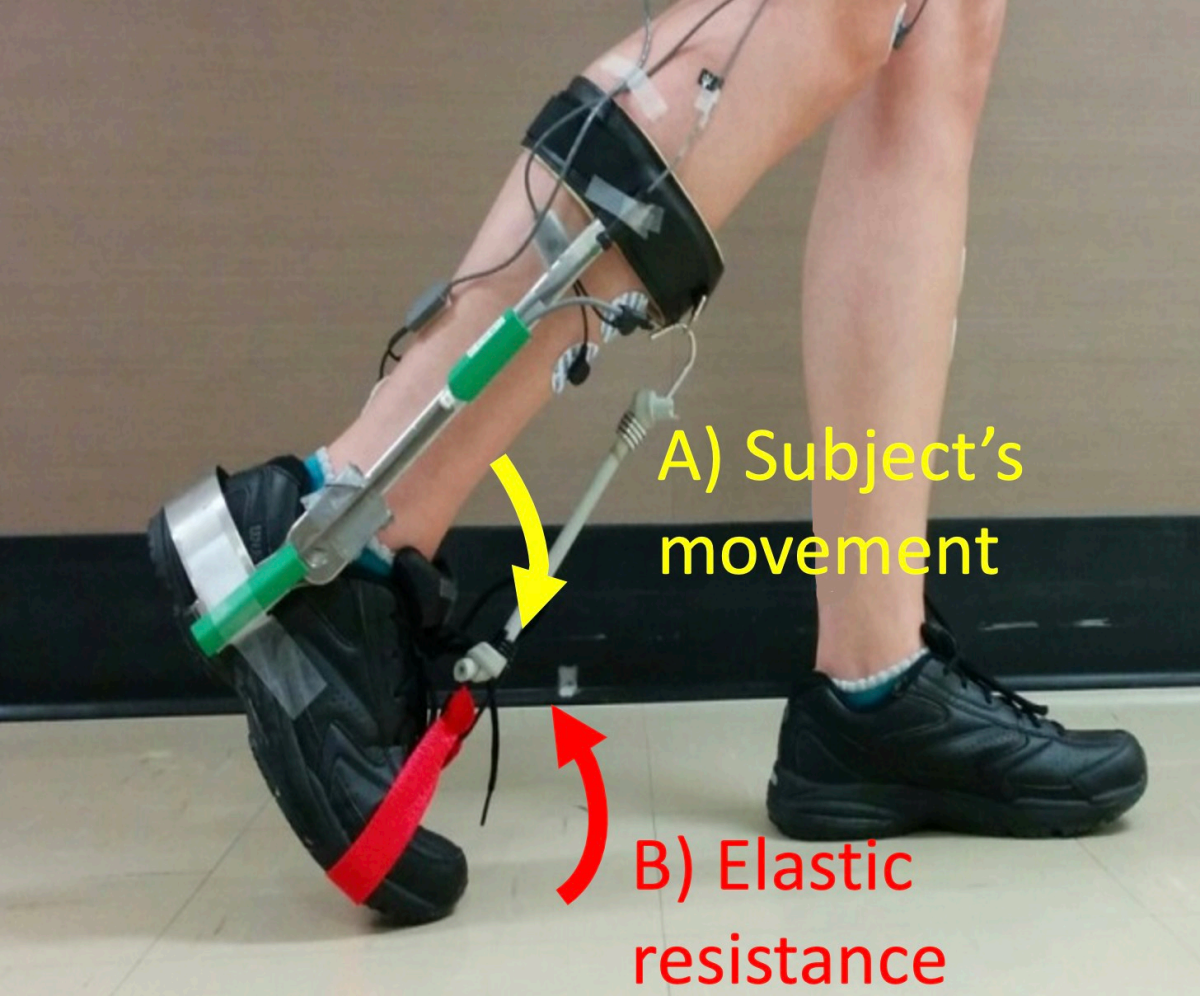
Picture of the modified AFO with the elastic force field on. A picture of the modified Klenzac ankle foot orthosis representing the subject’s movement (A) recorded by the electrogoniometer placed on the lateral stem of the modified AFO and the elastic resistance that participants had to resist (B).

Elastic tubing (10.5 cm-long; Thera-Band® Silver; The Hygenic Corporation, Akron, Ohio) was used to create a ‘force field’ that resisted plantarflexion. It was attached to the front of the modified AFO and to a strap at the level of the fifth metatarsal head (Fig 1). The elastic force perturbation pulled the foot upwards during swing and resisted push-off (max resistance reached at the end of push-off) but had little effect during the rest of the gait cycle (see Barthélemy et al., 2012 for more details [38]). The stiff lateral stems of the AFO ensured that the elastic tubing did not induce compression of the ankle joint.

### Protocol

A simple, low-cost push-off retraining protocol was developed using an elastic resistance used to resist plantarflexion, thus retraining push-off. Participants had to walk at self-selected speed overground, while voluntarily increasing their descending output to overcome the resistance applied on a modified AFO.

Participants walked overground in an 80-meter-long corridor. During each of 3 walking conditions, they wore a modified Klenzac ankle foot orthosis (AFO) on their right leg, on which elastic tubing was attached only during the second condition (Fig 1). The first condition (BASELINE) consisted of walking 4x80 meters in the corridor and served to characterize baseline gait parameters. During the second condition (EXPOSURE; 4x80 meters), Theraband Silver elastic tubing (Fig 1) was attached to the modified AFO to create a resistance against ankle plantarflexion. The lateral stems of the AFO absorbed elastic tubing tension, and participants therefore only felt a resistance to angular movement, with no joint compression. They were instructed to “*push against the elastic to overcome its resistance*”. The last condition (POST-EXPOSURE; 8x80 meters) was used to measure the persistence of the gait pattern modifications induced by training, and their duration following elastic removal. In order to capture the majority of possible aftereffects and to serve as a wash-out period, the POST-EXPOSURE recording session lasted twice as long as the other two conditions. Each participant chose their preferred gait speed during BASELINE. An experimenter specialized in clinical gait analysis walked slightly behind them during the 3 conditions and, during EXPOSURE and POST-EXPOSURE, systematically provided verbal feedback every 40 meters to minimize major gait deviations and reminded participants to push against the elastic tubing.

### Recordings

Relative ankle joint angles were recorded bilaterally and continuously during each walking condition using electrogoniometers (Biometrics Ltd., Ladysmith, Virginia). Data was transmitted to a desktop computer using wireless communication (Norangle; Noraxon USA inc., Scottsdale, Arizona) and saved at 1000 Hz/channel.

### Data processing

To minimize the amount of equipment worn by participants, individual gait cycles were identified from the continuous recordings using ankle angular velocity rather than foot switches. Gait cycles were aligned on peak plantarflexion angular velocity (an event occurring near toe-off) and a 500 ms window centered around this time was used for data extraction using a custom-made MatLab program (non-published method validated by visual inspection of the results). For each gait cycle, peak ankle plantarflexion angle and angular velocity (referred to as velocity throughout the article) were quantified. A step-by-step time course for these variables was then plotted together with a 95% confidence interval (95%CI) calculated from the mean of the last 50 baseline gait cycles.

### Statistics

To quantify the presence of motor adaptation during elastic exposure, and aftereffects after elastic removal (main study objective), the main variables were evaluated using 2 complementary methods: a time course analysis and a time point analysis.

Time course analysis was performed graphically using the confidence intervals [39], as previously described in Fortin et al. [29]. Briefly, an 11-points moving average was calculated for the Exposure and Post-Exposure data. The number of consecutive strides outside of the 95%CI was counted and used to quantify the presence and duration of changes.

For time point analysis, the following 5 epochs were defined:

- “Baseline late”: mean of the last 50 strides of the BASELINE period;
- “Exposure early”: mean of the first 5 strides of the EXPOSURE period;
- “Exposure late”: mean of the last 50 strides of the EXPOSURE period;
- “Post-exposure early”: mean of the first 5 strides of the POST-EXPOSURE period;
- “Post-exposure late”: mean of the last 50 strides of the POST-EXPOSURE period.

As data followed a normal distribution, a repeated measure ANOVA was applied separately to the difference in peak plantarflexion position and velocity, and for aftereffect durations. Level of significance was set at 0.05.

In the presence of the elastic, several motor strategies can be used to overcome the resistance: 1) returning to baseline ankle angle at push-off; 2) returning to baseline ankle push-off velocity; 3) a combination of 1) and 2). To identify which of these motor strategies might optimize retention over time (secondary study objective), a graph of individual relationship between changes in ankle position and changes in ankle velocity was produced using the following formula: 100* (Exposure late–Baseline late)/Baseline late. A Pearson correlation was used to determine if the slope was significantly different from zero and three zones were arbitrarily added to the graph (±20% of angle change; see Results).

## Results

### Ankle kinematics

**Fig 2:**
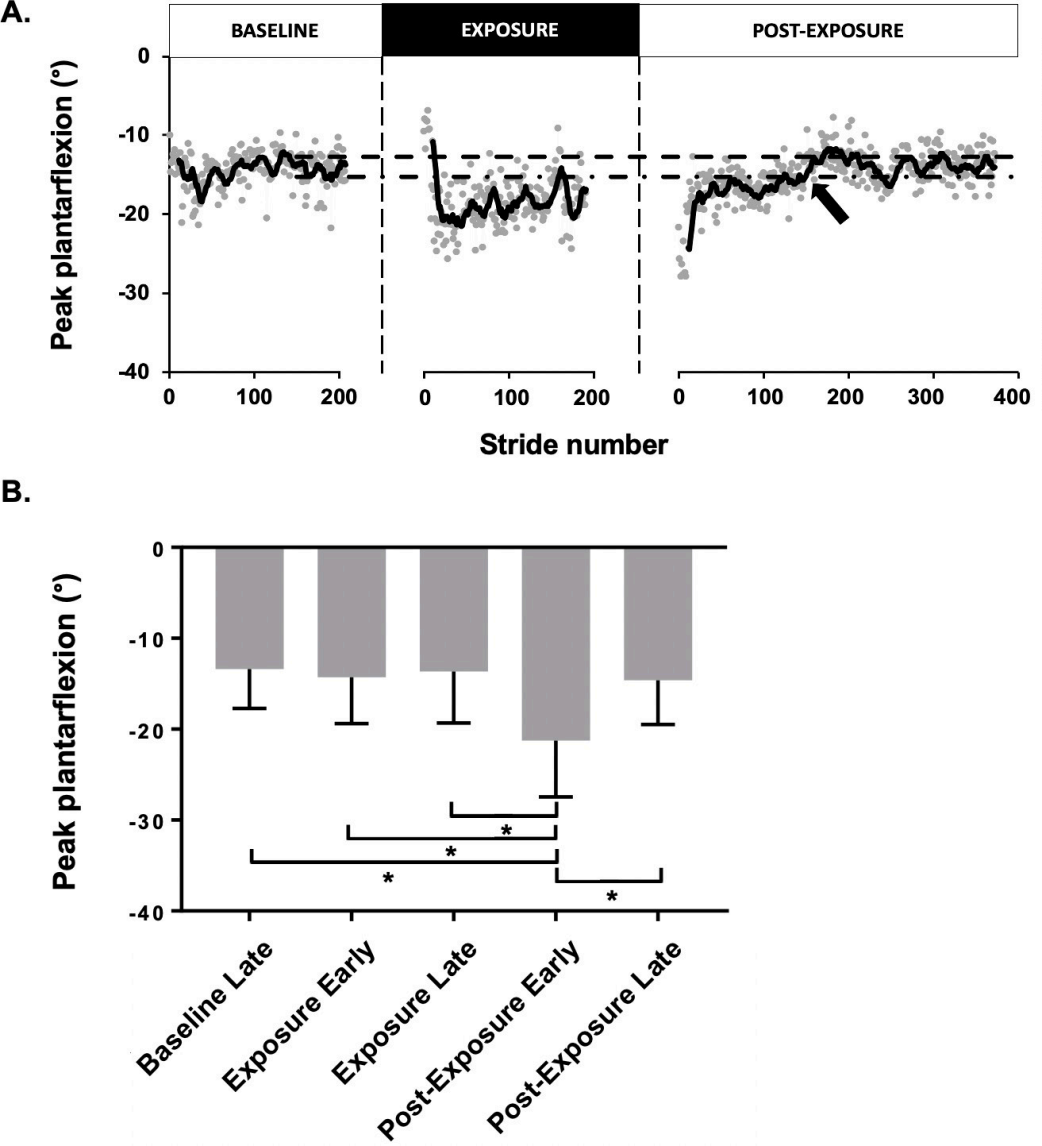
Effect of the force field on the peak plantarflexion angle. (A) Time course for one representative participant of the peak plantarflexion angle (°) during the three walking conditions. Each dot represents a single gait cycle. The 95% lower and upper confidence interval (dashed lines) is based on the mean of the moving average of the last 50 gait cycles peak plantarflexion from the post-exposure late. The black arrow indicates when the PF angle has returned to normal values (95% lower CI). (B) Means of the peak plantar flexion angle (°) for the group. Asterisk, P < 0.0001.

Fig 2A shows the peak plantarflexion time course of a representative participant. During the BASELINE condition, baseline late peak plantarflexion angle was 14.6±2.3°. With the elastic, peak plantarflexion angle was initially reduced to 9.2±1.6° (exposure early; p<0.05). The participant then gradually adapted to the force field by increasing peak plantarflexion to 17.7±3.1°. Upon elastic removal, there was a significant overshoot in peak plantarflexion to 25.9±2.5° (p<0.05) that gradually returned to baseline values in post-exposure late (13.9±1.9°; p>0.05). Using a graphical time course analysis, these aftereffects lasted 148 strides for this participant.

#### Group effects on peak plantarflexion angle

As a group, ANOVA results were statistically significant (p<0.0001, F (4, 29) = 20.91, R^2^ = 0.42). Participants showed a statistically significant increase in peak plantarflexion, from 13.4±4.2° in baseline late to 20.0±6.4° in post-exposure early (p<0.0001) for the right lower limb (Fig 2B). Their mean aftereffects duration was 150±156.7 strides (range: 0–462). They returned to 14.5±4.7° in post-exposure late, a value not statistically different from baseline. Moreover, when looking at the exposure early vs. late, the peak plantarflexion varies from 14.0±4.9° to 13.1±5.4°, which is not statistically different (p>0.05).

Regarding the contralateral leg, while small differences in peak plantarflexion were only found during exposure (20.8±4.2° in baseline late and 22.6±4.5° in exposure late; p = 0.002), no significant aftereffects were measured. This suggests that no adaptation occurred on the contralateral side.

#### Group effects on peak plantarflexion angular velocity

In addition to changes in peak plantarflexion angle, there was a significant change in plantarflexion velocity (p<0.0001, F (4, 29) = 15.30, R^2^ = 0.35), for the right (experimental) lower limb (Table 2). When baseline late was compared to post-exposure early (p<0.0001), going from 145.8±22.7°/s to 174.4±31.0°/s. On the other hand, post-exposure late velocity was not statistically different from baseline values (p>0.05).

**Table 2:** Group means for the kinematic values.

	Right Lower Limb (Trained)				
	Baseline Late	Exposure Early	Exposure Late	Post. Early	Post. Late
Peak PF ± SD (°)	13.4 ± 4.2	14.0 ± 4.9	13.1 ± 5.4	20.0 ± 6.4	14.5 ± 4.7
Significance	p<0.0001, F (4, 29) = 20.91, R^2^ = 0.42				
Peak Angular Velocity ± SD (°/s)	145.8 ± 22.7	147.2 ± 33.6	139.4 ± 31.0	174.4 ± 37.4	150.4 ± 24.0
Significance	p<0.0001, F (4, 29) = 15.30, R^2^ = 0.35				
AE Range (Baseline)	-	-	-	0 to 462 strides	
AE Range (Post. Late)	-	-	-	0 to 301 strides	

The table shows the peak plantarflexion (Peak PF) mean values for the group, with its standard deviations (SD). The peak plantarflexion velocity with its standard deviations is also shown. ANOVA results for the peak plantarflexion angle and angular velocity are presented below each variable. The aftereffect range (AE range) is presented for each way of calculating it: with the 11-points moving average calculated from the 50 last strides of the baseline condition and from the post-exposure condition. For the left lower limb, no significant changes were observed regarding peak plantarflexion angular velocity.

### Aftereffects duration variability across participants

As mentioned above, the duration of aftereffects ranged from 0 to 462 in the group, with a mean duration of 150±156.7 strides, showing a large variability across participants. This is, however, when the aftereffects duration is calculated based on baseline late data. As presented in Table 2, this variability is reduced when aftereffects duration is measured relative to the 50 last strides of the post-exposure condition. In this case, mean aftereffects duration is 80.6±91.2 strides, ranging from 0 to 301 strides.

**Fig 3:**
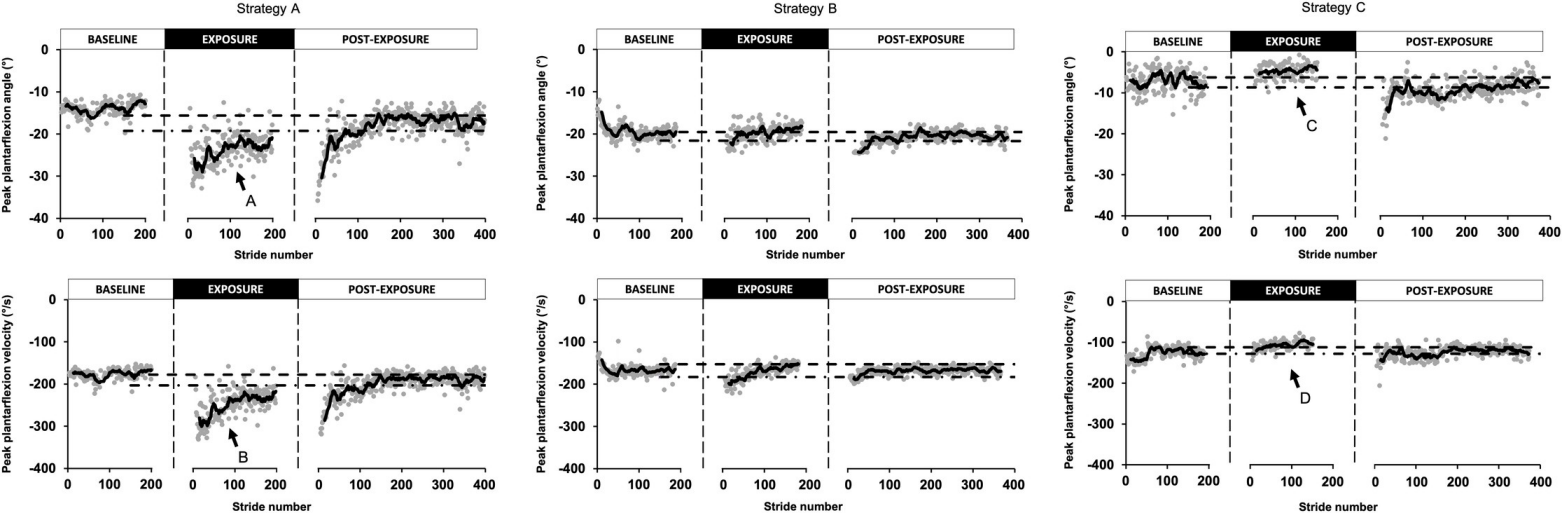
Examples of adaptive strategies. This figure represents the three adaptive strategies found during the exposure condition in our participants. For each column, the peak plantarflexion angle and the peak plantarflexion velocity is shown for a representative participant. Strategy A represents a participant with an increased peak plantarflexion angle and velocity maintained through the whole condition. Strategy B represents a participant that increased its plantarflexion velocity at the very beginning of the condition without maintaining this increase. Strategy C represents a participant that decreased its plantarflexion angle and velocity during the exposure condition.

However, even with this “correction”, a large variability remains. Detailed analysis of individual adaptation time courses revealed different strategies across participants. Three representative examples are presented in Fig 3. Participants represented in the left column (strategy A) showed the longest aftereffect durations. Looking at their differences with baseline in peak ankle plantarflexion angle and peak plantarflexion velocity, these participants actually pushed more (increased peak plantarflexion angle compared to baseline; *arrow A*) and faster (increased peak plantarflexion velocity compared to baseline; *arrow B*) in the presence of elastic resistance than during baseline walking. On the contrary, participants represented in the right column (strategy C) had the least aftereffects. These participants decreased their peak push-off angle (*arrow C*) and velocity (*arrow D*) relative to baseline. Finally, the participants represented in the centre column (strategy B) either increased their push-off velocity OR increased plantarflexion angle while maintaining the other variable around the baseline value. They had intermediate aftereffect durations.

**Fig 4:**
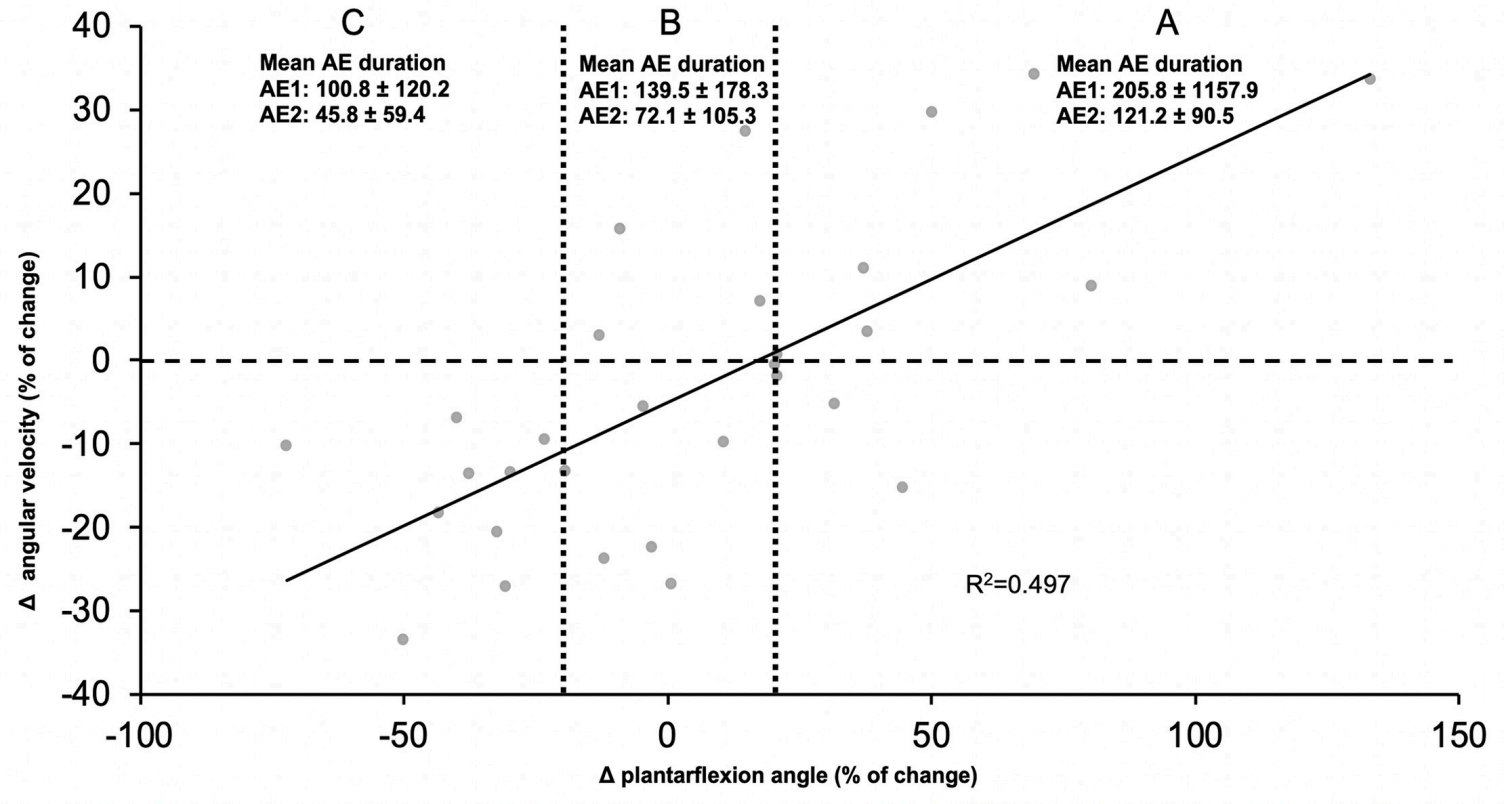
Continuum of all participants in regard of their strategies. The X axis shows the difference in position, while the Y axis shows the difference for the push-off velocity, represented as percentage of change. Zone A represents a change of more than 20% in plantar flexion, zone B a change of ±20% in plantarflexion and zone C represents a change of more than -20% in plantar flexion. In each zone, the mean aftereffect duration is presented for aftereffects calculated from the baseline (AE1) and from the post-exposure late (AE2).

The relationship between individual strategies and aftereffect duration is presented for the whole group in Fig 4. The latter plots the difference in velocity as a function of the difference in ankle position with associated aftereffect duration in the label below each strategy. Each participant is represented by a point on the graph. On the X axis, the difference in peak plantarflexion angle is represented by the exposure late values minus the baseline late values, while on the Y axis, the same difference is shown for plantarflexion (push-off) velocity. As shown on the graph, the R^2^ value is 0.497. On this figure, it is possible to see that individual strategies are found as a continuum between the three examples presented in Fig 3: one in the upper-right quadrant (strategy A), one around the centre of the graph (strategy B) and the last one in the lower-left quadrant (strategy C).

### Relation between kinematic parameters

The curve fitting for the linear regression presented on Fig 4 was statistically different from 0 (p<0.0001). When looking at the repeated measure ANOVAs between the three zones determined on the graph (zone A: [-100; -20], zone B: [-20; 20], zone C: [20; 150]), there is a significant difference between groups A-C (p<0.002) and B-C (p<0.05) for the difference in speed and for the aftereffect duration (post-exposure late).

## Discussion

The main goal of this study was to determine if a motor adaptation protocol similar to those used for dorsiflexor muscles could be adapted to retrain ankle plantarflexors during push-off. By comparing ankle kinematics activity before, during and after exposure to an elastic force field applied during walking ***overground***, our results suggest that the neural control of soleus can indeed be modified if training is performed under specific conditions.

### Aftereffects following the elastic removal

Upon removing the elastic resistance, participants showed increased peak plantarflexion angle and velocity that were maintained over several gait cycles. These results are equivalent to those presented after TA adaptation [17,40]. Therefore, the presence of aftereffects after the elastic removal supports the hypothesis that it is possible to modify the feedforward (central) command controlling ankle plantarflexors during push-off. Similar modifications in feedforward command were demonstrated at other joints previously [28,35,41–43], but initial work at the ankle during push-off had previously been unsuccessful [27]. The mean duration of this aftereffect was quite long on average (180 cycles) when compared to TA adaptation (20 cycles; [40]).

This increased plantarflexion during *post-exposure* is providing further evidence that the aftereffects resulted from a continued use of the adapted motor pattern, and not just from a change in the way participants walked.

### Adaptive strategies

Figs 3 and 4 together suggest that for push-off adaptation, only looking at peak plantarflexion angular position changes might not be sensitive enough to capture the adapted motor strategy used by our participants. Adding information about push-off velocity helps predicting if a participant is going to show large aftereffects or not. Indeed, when participants increased both peak plantarflexion angle AND peak plantarflexion velocity during push-off, they showed longer aftereffects than if either one of these strategies was used alone. This finding suggests that the explosive contraction aspect measured by push-off velocity could be very important for a more complete adaptation during push-off. This explosive aspect has been described previously as the ability to use the muscles’ torque-producing capacity explosively and tend to be more conductive to explosive performance with concentric contractions [44]. As walking while resisting an elastic force field uses concentric contraction of ankle plantarflexors, this might explain the importance of the explosive aspect of the muscle contraction to predict responders. When designing force field adaptation protocols for the rehabilitation setting, it might therefore be very important to insist, by giving clear instructions to participants, on increasing push-off velocity.

### Effects on the contralateral leg

Regarding the contralateral leg, only a small and transient change in peak plantarflexion angle was observed *during* the exposure condition, with no carry-over to post-exposure. Push-off velocity, however, was not affected by the elastic force field training. This suggests that the motor learning process is mainly specific to the trained leg. Previous studies using split-belt training with stroke also suggest that training will mainly improve the trained (affected) side [45,46].

### Ecological validity of the training environment and clinical implications

To our knowledge, this study is the first to report the ability to use force field adaptation to train plantarflexor muscles to produce more activity during push-off.

The fact that aftereffects consisting of increased plantarflexion were measured shows that even muscles with a strong **positive** feedback component such as ankle plantarflexors to their motor output are amendable to environmentally-driven plastic modifications in central drive. Such capacity therefore has the potential of being tapped into for the design of future neurorehabilitation protocols using phase and task-specific resistive training, such as can be delivered using robotized gait orthoses.

The increase in muscle activation will not only increase the number of motor units recruited, but in the long run, if this protocol is repeated over several sessions, it might, in addition, lead to structural modifications in muscle fibers, similar to strength training, thereby increasing muscle strength and muscle mass, two additional beneficial factors for rehabilitation. By increasing the strength of the muscles responsible for push-off, it might be possible to increase gait speed of patients. It is well known that lower limb strength is correlated to gait speed [4,25,26]. Thus, by increasing their gait speed, it would be possible for patients to meet the minimal requirement to be functional in society. Moreover, in chronic stroke patients, it has been shown that task-related training circuits focusing on functional activities improve performance at locomotor tasks [14]. As this protocol involves walking overground while resisting the elastic, it could be easily integrated in activities of daily living, or into circuit training during rehabilitation. Finally, as discussed in Blanchette *et al*. [40], an increase of 5° in dorsiflexion range in a population of persons with a neuromuscular disease is considered to be clinically significant. In this study, we have shown an increase of almost 7° towards plantarflexion in the experimental leg. This could mean that this protocol could possibly significantly improve the gait pattern of people living with a neurological impairment, while being specific to the leg you want to train. Further studies in clinical populations are required to better understand the functional implications of this modification in the gait cycle with regard to the overall gait pattern. Based on our findings, we would recommend that such resistance training in further studies or in clinical settings should focus on the impaired leg to maximise improvement.

### Strengths and limitations of the study

This study has some limitations. First, kinematic recordings were made only at the ankle. It is therefore possible that additional modifications in the gait pattern occurring at other joints were not quantified. A second limitation is the absence of kinetic variables. It would be interesting to record the anteroposterior ground reaction forces in future studies in order to validate the efficiency of the modification in the gait pattern. It would also have been interesting to compare gait speed before and after the exposure phase or during the exposure phase to ensure constant gait speed. As push-off training should improve gait speed, objectifying the magnitude of this effect would be clinically useful.

This study also has several strengths. this approach, using an AFO orthosis and elastic tubing, requires little materials and is cheap to implement, thereby facilitating its potential use in the clinic.

## Conclusion

These results show that it is possible to retrain push-off while walking overground with a protocol similar to that previously used for dorsiflexor training. This training results in increased peak plantarflexion angle and velocity in the majority of participants *in a time period too short for changes in muscle structure to occur*. The next step will be to test this protocol in neurological populations that have impaired push-off control, to objectify the presence of aftereffects and measure the generalizability of this approach as a potential intervention for gait neuro-rehabilitation.

## Supporting information

S1 File Dataset. (XLSX)
